# Retraction: Terminal drought and heat stress alter physiological and biochemical attributes in flag leaf of bread wheat

**DOI:** 10.1371/journal.pone.0284070

**Published:** 2023-04-19

**Authors:** The PLOS ONE Editors

The *PLOS ONE* Editors retract this article [1] because it was identified as one of a series of submissions for which we have concerns about authorship, competing interests, and peer review. We regret that the issues were not addressed prior to the article’s publication.

MAC and SUA did not agree with the retraction. All other authors either did not respond directly or could not be reached.
